# Synthetically trained convolutional neural networks for time-resolved aortic segmentation of four-dimensional flow magnetic resonance imaging

**DOI:** 10.1016/j.jocmr.2026.102735

**Published:** 2026-04-21

**Authors:** Gloria Wolkerstorfer, Pietro Dirix, Cosima Jahnke, Ingo Paetsch, Robert Manka, Stefano Buoso, Sebastian Kozerke

**Affiliations:** a Institute for Biomedical Engineering, University Zurich and ETH Zurich, Zurich, Switzerland; b Department of Cardiology and Electrophysiology, Heart Center Leipzig at University of Leipzig, Leipzig, Germany; c Diagnostic and Interventional Radiology, Department of Cardiology, University Hospital Zurich, Zurich, Switzerland

**Keywords:** Cardiovascular magnetic resonance, 4D flow MRI, Image segmentation, Convolutional neural network, Image synthesis, Time-resolved aortic segmentation

## Abstract

**Background:**

Supervised learning-based approaches are increasingly used for vessel segmentation in four-dimensional (4D) flow cardiovascular magnetic resonance (CMR). However, their widespread adoption is challenged by the need for diverse, reliably annotated datasets, sensitivity to acquisition and reconstruction settings, and the lack of fully defined ground truth.

**Methods:**

In this study, we investigated the use of realistic, fluid mechanics-informed synthetic 4D flow CMR data to train convolutional neural networks for time-resolved aortic segmentation. The availability of fully defined ground-truth vessel geometries in synthetic data enabled quantitative evaluation before evaluation on in vivo data acquired across three scanners with varying field strengths and protocols. Four training strategies were evaluated: one using in vivo data only (R-28) and three based on increasing amounts and diversity of synthetic data (S-28, S-multi, and S-full). Performance was assessed using voxel- and surface-based metrics, including Dice similarity coefficient (DSC), Hausdorff distance (HD), and Bland-Altman analysis.

**Results:**

The S-full model achieved the best performance on the in vivo test dataset, with a DSC of 0.956 ± 0.017 and a HD of (1.7 ± 1.5) mm, relative to reference annotations. Bland-Altman analysis of cross-sectional areas showed small biases and narrow limits of agreement, with 1.1% [−10.9, 13.1]% for the ascending aorta and −1.0% [−13.1, 11.1]% for the descending aorta. For the in vivo trained model (R-28) evaluated on synthetic data, relative cross-sectional area measurements yielded biases of 3.1% [−10.9, 17.0]% in the ascending aorta and 4.0% [−8.2, 16.2]% in the descending aorta, relative to synthetic ground truth.

**Conclusion:**

This work demonstrates that purely synthetic 4D flow CMR can be used to train neural networks for time-resolved aortic segmentation of in vivo 4D flow CMR data, enabling fully automatic inference and quantitative evaluation against fully defined ground truth.

## Graphical abstract

**Figure:**
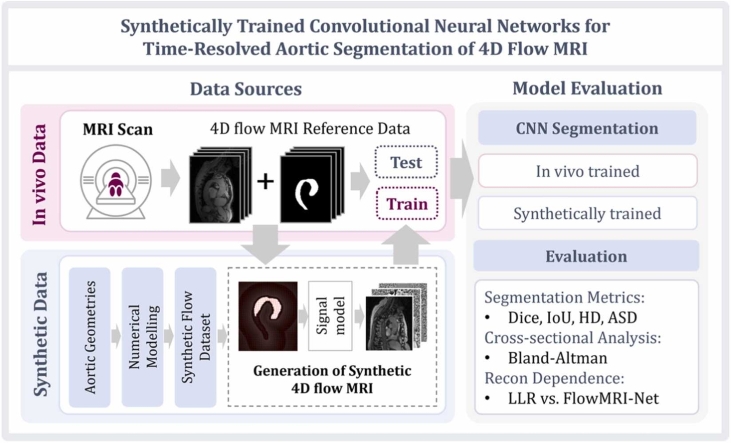

## 1 Introduction

Four-dimensional (4D) flow cardiovascular magnetic resonance (CMR) offers valuable insights into vascular anatomy and flow dynamics, enabling quantitative assessment of cardiovascular conditions [1], [2], [3]. Despite its clinical potential, broader adoption remains limited, partly due to the lack of robust, scalable, and widely available vessel segmentation pipelines.

Early approaches for 4D flow CMR segmentation predominantly relied on atlas-based registration techniques [4], [5]. While these approaches provide anatomically consistent segmentations (Dice ∼0.88 [6]) and can be advantageous in data-limited settings, they require dataset-specific calibration and computationally intensive optimization, limiting scalability in larger or heterogeneous cohorts. In addition, their dependence on initial segmentation masks imposes a substantial annotation burden and complicates propagation across the cardiac cycle.

More recently, deep learning-based methods, particularly convolutional neural networks (CNNs) with U-shaped architectures, have emerged as the state-of-the-art in medical image segmentation, offering improved computational efficiency and accuracy [6], [7], [8], [9], [10]. In aortic 4D flow CMR, CNN-based methods report Dice similarity coefficients (DSC) of 0.87 to 0.94 across contrast-enhanced [11], [12], [13] and non-contrast datasets [9], [14], [15], with peak performance up to 0.95 in single-phase (static) segmentation settings [16]. The self-configuring nnU-Net framework [17] further advances this field by automatically adapting preprocessing, architecture configuration, and training strategies, achieving similarly high performance (DSC of 0.95 [9], [18] for static aortic segmentation), without extensive manual tuning.

Most prior works focus on single-phase segmentations, obtained via phase-contrast magnetic resonance angiography (PC-MRA), rather than fully time-resolved volumetric data. Consequently, reported performance metrics are not directly comparable between single-phase and time-resolved methods. While single-phase segmentation is well established, time-resolved aortic segmentation remains relatively unexplored, with only a few studies reporting DSC of 0.89 to 0.93 [11], [12], [13] on contrast-enhanced data. This limitation restricts the applicability of existing methods in capturing dynamic vessel motion and time-resolved hemodynamic analysis.

Recently, Merton et al. [7] demonstrated the feasibility of time-resolved aortic segmentation using nnU-Net on magnitude-based balanced steady-state free precession CMR data, achieving a DSC of 0.93 ± 0.02 in a cohort of 14 subjects (10 for training, 4 for testing). Notably, each subject contributed three scans with annotations of two cardiac phases, resulting in a total of 84 manual segmentations.

Despite these advances, robust generalization of deep learning models across centers remains a major challenge. Multi-center studies have shown reliable generalization with DSC between 0.90 and 0.95 on static non-contrast-enhanced segmentation, but typically require large and diverse datasets, comprising several hundred subjects [14], [16]. However, the assembly of such datasets is challenged by data-sharing restrictions, privacy constraints, and the considerable manual annotation effort. Consequently, the development of robust and generalizable segmentation models across institutions remains limited. Although multi-vendor studies and comprehensive processing pipelines have been reported [14], [19], the restrictions on model access impede reproducibility and broader translation.

Synthetic data generation has emerged as a promising strategy to address these challenges by enabling scalable dataset expansion while avoiding privacy constraints [20], [21], [22]. Prior work has shown that synthetic PC-MRA data can augment small in vivo datasets and improve static aortic segmentation performance [22]. However, synthetic data were used only as an additive component to real (in vivo) data, and the approach was limited to static segmentation tasks, with no public dataset or models released. Whether models trained exclusively on synthetic data can generalize across heterogeneous imaging settings, particularly for time-resolved volumetric segmentation, remains to be demonstrated.

Recently, Dirix et al. [23] introduced a fluid mechanics-informed framework for generating synthetic 4D flow CMR data of the thoracic aorta by combining realistic anatomical geometries [20], [24] with pulsatile flow simulations. These synthetic datasets provide fully defined ground-truth geometries and flow fields, and can be embedded into in vivo MRI backgrounds, enabling controlled evaluation of segmentation performance, and are publicly available.

In this work, we investigate whether models trained exclusively on synthetic 4D flow CMR data can achieve robust, time-resolved segmentation of the thoracic aorta in heterogeneous in vivo datasets. Using the nnU-Net framework, we systematically compare models trained on synthetic-only and in vivo-only datasets and evaluate performance on multi-scanner data acquired across varying field strengths and acquisition protocols. By releasing trained models and example data, we aim to promote transparency, reproducibility, and broader adoption.

## 2 Methods

**Fig. 1:**
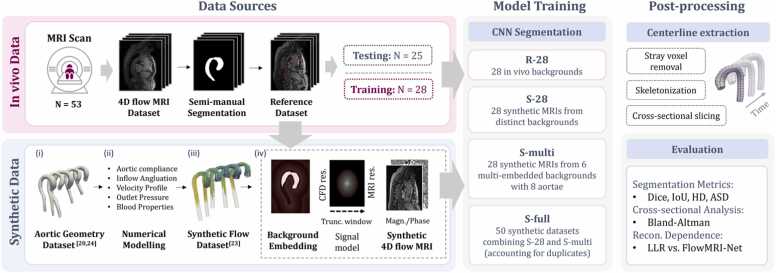
Overview of data sources, model training and analysis. In vivo 4D flow MRI data (top left) were acquired across three sites. Synthetic datasets were generated by embedding CFD-simulated aortic flow and geometries into in vivo backgrounds (bottom left, (i)-(iv)). Segmentation models were trained using the nnU-Net framework on in vivo and synthetic datasets (middle). Post-processing included centerline extraction and quantitative evaluation using voxel- and surface-based metrics (right). *4D* four-dimensional, *MRI* magnetic resonance imaging, *CFD* computational fluid dynamics, *CNN* convolutional neural network, *DSC* Dice scores, *HD* Hausdorff distance, *ASD* average surface distance, *IoU* intersection over union, *LLR* locally low-rank regularization.

An overview of our approach is presented in Fig. 1. In vivo 4D flow CMR data were acquired from healthy subjects and patients across three sites (Section 2.1). Synthetic datasets were generated by embedding computational fluid dynamics (CFD)-simulated aortic geometries and flow fields into a subset of in vivo background images (Section 2.2), resulting in realistic 4D flow CMR data with fully defined ground-truth geometry and flow.

Four segmentation models were trained using the nnU-Net framework on distinct datasets: one based on in vivo data only (R-28) and three based on synthetic data with varying levels of diversity (S-28, S-multi, and S-full). Post-processing included centerline extraction, cross-sectional radius estimation, and quantitative evaluation using voxel- and surface-based metrics.

### 2.1 In vivo cohort

Data were acquired across three sites using 1.5T (sites B and C) and 3.0T (site A) MR systems (Philips Healthcare, Best, the Netherlands). All subjects provided written informed consent, and data collection complied with institutional and ethical guidelines. The entire study cohort comprised a total of 53 subjects, with 33 subjects from site A (6 healthy volunteers and 27 patients with aortic stenosis), 10 healthy subjects from site B, and 10 subjects from site C, scheduled for atrial flutter ablation. While data from sites A and B were acquired natively, contrast-enhanced 4D flow CMR data were obtained from site C.

Acquisition parameters included spatial resolution 2.5 × 2.5 × 2.5 mm³, field of view 229–360 × 258–402 × 60–90 mm³, temporal resolution 45–60 ms, flip angle 8°–15°, repetition time (TR) 4.8–5.1 ms, echo time (TE) 2.7–3.4 ms, and velocity encoding (VENC) 150–450 cm/s. VENC values were adapted based on reference two-dimesional flow MRI measurements, with the higher values applied in subjects with aortic valve stenosis.

At 3T, data were collected with a 13-point VENC scheme using VENC settings of 50/250 cm/s for healthy (N = 6, 2 female) and 150/350 cm/s for aortic stenosis subjects (N = 27, 8 female). At 1.5T, data were acquired with a 4-point scheme using a VENC of 150 cm/s in all directions. Free-breathing data were acquired using pseudo-spiral Cartesian undersampling (acceleration factor R = 4–6.7) with repetitive sampling of k_y_ = k_z_ = 0 profiles for respiratory binning [25]. A summary of the study cohort, including train and test data split, is outlined in Table 1.

**Table 1:** Study cohort overview: acquisition and reconstruction parameters, age range, and train and test split.

	Site A	Site B	Site C
Field strength	3T	1.5T	1.5T
Contrast administration	No	No	Gadolinium
#Training/#Test	28/5	-/10	-/10
Age/range	75±13	39±14	62±14
Male/female	23/10	5/5	5/5
Image reconstruction	LLR	LLR, FlowMRI-Net	LLR

*LLR* locally low-rank regularization

Image reconstruction was performed following MR data-driven respiratory binning into three equally populated bins [26], retaining only the expiratory state for analysis. Accordingly, the effective acceleration factor for the end-expiratory bin ranged from 12–20. For image reconstruction, we employed locally low-rank regularization (LLR) [27], supplemented with Bayesian unfolding [28] for the 13-point encoded data from site A, as well as a self-supervised learning approach (FlowMRI-Net) [29]. For the 10 cases acquired at site B, images were reconstructed using both LLR and FlowMRI-Net to assess the sensitivity of the segmentation network to different reconstruction approaches.

**Fig. 2:**
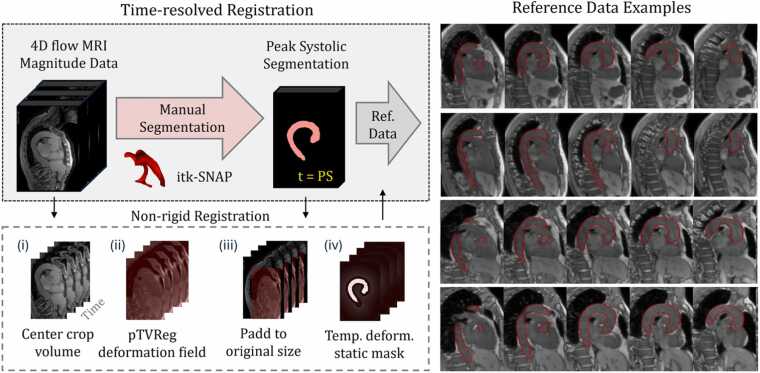
Generation of reference segmentation masks via non-rigid registration. Manual segmentations were created using ITK-SNAP. Temporal deformation fields, computed from magnitude images after cropping, were applied to propagate the initial static segmentation across the cardiac cycle. Four representative subjects are shown on the right, with contours in red. *4D* four-dimensional, *MRI* magnetic resonance imaging.

Reference segmentations of the thoracic aorta (from the sinotubular junction to the proximal abdominal aorta) were created manually on magnitude images at peak systole by two experienced annotators using ITK-SNAP [30]. Segmentations were initially generated by one experienced annotator and subsequently reviewed and refined by a second annotator. The brachiocephalic, left common carotid, and left subclavian arteries were not included. Temporal registration across the cardiac cycle was performed using the non-rigid registration package pTVReg [31], see Fig. 2 (left). As registration was used to generate reference segmentations, it does not constitute an independent baseline for comparison and was therefore not evaluated separately. In our study, propagation of reference segmentations across the cardiac cycle required approximately 40–60 min per subject using registration-based propagation. On the right of Fig. 2, four exemplary reference segmentations generated using this approach are shown. All segmentation masks were manually inspected by an expert and corrected when necessary, providing reference annotations for evaluation. For subjects from site B, reference annotations were generated separately for each reconstruction approach.

### 2.2 Synthetic cohort

The synthetic cohort was generated according to the procedure described previously [23], which combines CFD simulations of healthy and stenosed aortic geometries with in vivo 4D flow CMR backgrounds. The approach is schematically presented in Fig. 1 (bottom left). For each generated case, an aortic geometry is sampled from a distribution defined by a statistical shape model derived from two independent datasets comprising 25 healthy geometries [24] and 26 pathological geometries [20], respectively. Pulsatile velocity profiles were prescribed at the inlet (representing either healthy or stenosed valve conditions). Subsequently, CFD simulations were performed to calculate time-resolved velocity vector fields. After conversion of the velocity vector fields into MR signals, the resulting synthetic aortic simulation was embedded into the in vivo 4D flow CMR backgrounds using a dual-registration approach. First, a rigid alignment between the in-silico aorta with the aortic segmentation of a selected in vivo background was performed using coherent point drift approach (pycpd [32]). Second, non-rigid registration (pTVReg [31]) was used to match the background with the synthetically simulated aorta. This two-step approach ensured smooth deformations while preserving realistic integration of flow and anatomy.

Synthetic data were generated from 28 in vivo subjects from site A, each paired with a realistic CFD-simulated aortic geometry including dynamic wall motion and corresponding flow fields, forming the S-28 dataset. To increase anatomical diversity, eight additional synthetic geometries were generated using the same modeling and simulation approach. Each geometry was embedded into multiple in vivo background images from six subjects included in S-28. This resulted in 28 additional synthetic datasets (S-multi). Although some background images were reused, all embedded aortic geometries were unique. After accounting for six overlapping combinations between the synthetic sets, the final synthetic cohort comprised 50 unique datasets (S-full).

### 2.3 Model training

#### 2.3.1 Train and test sets

For neural network training, 28 of the 33 subjects from site A were used. These correspond to the same cases employed for 4D flow CMR data synthesis. The test set consisted of the remaining 5 subjects from site A (2 healthy and 3 stenosed subjects), along with all subjects from sites B and C.

The nnU-Net framework [17] was trained on the following combinations: one in vivo dataset and three synthetic datasets. For the in vivo dataset, the original 28 subjects (from site A) with their native anatomical backgrounds were used, constituting the R-28 dataset. The resulting four models were then defined according to their training dataset names: 1) R-28, trained on 28 in vivo cases; 2) S-28, trained on 28 synthetic cases generated by embedding 28 CFD-simulated geometries into the R-28 backgrounds; 3) S-multi, trained on 28 synthetic cases generated from multiple combinations of eight CFD-simulated geometries embedded within six selected backgrounds; and 4) S-full, trained on the complete set of 50 unique synthetic datasets, by combining S-28 and S-multi while accounting for six overlapping cases.

The test dataset consisted of 25 in vivo subjects (site A: N = 5; site B: N = 10; and site C: N = 10).

#### 2.3.2 Data preparation and model training

Preprocessing included linear up-sampling by a factor of 2, central cropping and intensity normalization to the range [0,1]. The resulting volumes had dimensions of (244, 144, 64) per cardiac frame. Each cardiac frame was treated as an independent three-dimensional sample. The nnU-Net framework applies extensive data augmentation during training; therefore, no additional data augmentation was applied. Additional conventional augmentation strategies were evaluated but did not result in measurable performance improvements. All datasets were organized in the standard nnU-Net structure and processed through the framework’s pipeline. Segmentation was performed using magnitude data only. Each model was trained for 1000 epochs using the default nnU-Net loss function (combined Dice and cross-entropy loss) with a single fixed train-validation split.

### 2.4 Model evaluation

#### 2.4.1 Post-processing

Fig. 1 (right column) provides a schematic overview of the post-processing steps applied to the segmented volumes. Isolated regions below a predefined volume threshold were removed to eliminate outliers. Vessel centerlines were then derived via skeletonization (skan [33]) followed by B-spline interpolation. Before metric computation, segmentations were clipped at the inlet and outlet to match the anatomical extent of the reference masks. This ensured consistent evaluation boundaries, as the reference annotations excluded the aortic root.

#### 2.4.2 Segmentation metrics

For each time frame, segmentation performance across the four models was evaluated relative to the reference segmentation of the test dataset (Section 2.3.1) using DSC, Intersection over Union (IoU), Accuracy (Acc), Precision (Prec), and Recall (Rec). In addition, the Hausdorff distance (HD) and average surface distance (ASD) were computed between the predicted and reference aortic surfaces. A definition of all segmentation metrics is outlined in the Appendix. Statistical differences between models were assessed using the Friedman test for paired comparisons on time-averaged data, followed by pairwise Wilcoxon signed-rank tests with Benjamini-Hochberg false discovery rate (FDR) correction. Adjusted p-values are reported as p_FDR_.

#### 2.4.3 Best-performing model evaluation

For the S-full model, several additional analyses were performed on the test dataset from sites A, B, and C. Binary masks were first converted to triangulated surface geometries using Pyvista [34], from which 23 equidistant cross-sections were extracted along the aorta. These time-resolved cross-sections were then used to compute radius variations along the centerline and to quantify relative radius errors with respect to reference values. Therefore, the radius was computed as the mean Euclidean distance from all contour points of a given cross-section to its center of mass. Additionally, agreement in cross-sectional area at the pulmonary artery level (PA-level) was assessed from the binary masks using Bland-Altman analysis, with limits of agreement (LOA) and 95% confidence intervals (CI). Relative area change across cardiac phases was assessed with results stratified into three age groups: younger than 45 years (N = 9), 45 to 70 years (N = 8), and older than 70 years (N = 8, including 2 stenosed cases). Lastly, robustness to reconstruction methodology was evaluated using the 10 subjects from site B, for whom both LLR and FlowMRI-Net reconstructions were available. Additional evaluation was performed by retrospectively undersampling the data to acceleration factors of 16 and 20 and reconstructing them using FlowMRI-Net followed by quantifying and comparing segmentation performance.

## 3 Results

### 3.1 Model comparison

**Fig. 3:**
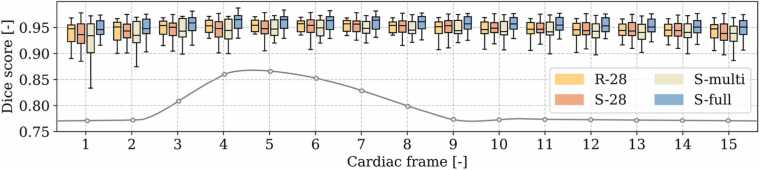
Time-resolved Dice statistics for the four segmentation models on the in vivo test dataset. Boxplots are shown for the first 15 cardiac frames for the in vivo (R-28, yellow), synthetic (S-28, orange), multi-embedded synthetic (S-multi, green), and full synthetic (S-full, blue) models. The gray line illustrates an example flow evolution.

Time-resolved Dice scores for the four models, R-28 (in vivo, yellow), S-28 (synthetic, orange), S-multi (multi-embedded synthetic, green), and S-full (all synthetic combined, blue), are presented in Fig. 3, with boxplots shown alongside an example flow curve in gray. Due to varying temporal resolutions across subjects (Section 2.1), the first 15 cardiac frames are shown. All models maintained high segmentation accuracy throughout the cardiac cycle, including diastolic frames. The S-full model consistently demonstrated higher median Dice values compared to the other models.

For statistical analysis, segmentation metrics were temporally averaged per subject across all available cardiac frames (Table 2). Overall, the S-full model achieved the highest performance (DSC: (95.6 ± 1.7)%; HD: (1.7 ± 1.5) mm), followed by comparable performance of the S-28 (DSC: (94.6 ± 2.1)%; HD: (2.7 ± 4.2) mm) and the R-28 model (DSC: (94.7 ± 1.7)%; HD: (2.2 ± 3.0) mm). The S-multi model showed the lowest performance with greater variability (DSC: (92.9 ± 8.4)%; HD: (5.8 ± 11.6) mm).

**Table 2:** Temporally averaged distance metrics on the in vivo test dataset for the R-28, S-28, S-multi, and S-full models.

Model	DSC ↑	IoU ↑	Acc ↑	Prec ↑	Rec ↑	HD (mm) ↓	ASD (mm) ↓
R-28	0.947±0.017	0.900±0.030	0.997±0.002	0.942±0.030	0.953±0.024	2.2±3.0	0.7±0.3
S-28	0.946±0.021	0.898±0.037	0.997±0.002	0.943±0.026	0.950±0.037	2.7±4.2	0.7±0.4
S-multi	0.929±0.084	0.874±0.097	0.996±0.003	0.928±0.084	0.933±0.096	5.8±11.6	1.1±1.5
S-full	**0.956±0.017**	**0.915±0.031**	**0.997±0.002**	**0.952±0.025**	**0.960±0.025**	**1.7±1.5**	**0.6±0.2**

*DSC* Dice similarity coefficient, *IoU* intersection over union, *Acc* accuracy, *Prec* precision, *Rec* recall, *HD* Hausdorff distance, *ASD* average surface distance

A Friedman test revealed significant overall differences between models across voxel- and surface-based metrics, including Dice, IoU, Accuracy, Precision, HD, and ASD (all p < 0.01), while Recall did not reach statistical significance (p = 0.057). Post-hoc paired Wilcoxon tests with FDR correction showed that the S-full model significantly outperformed R-28 in Dice (median difference 0.009, 95% CI [0.005–0.014], p_FDR_ = 0.003) and HD (median difference 0.21 mm, p_FDR_ = 0.052) with a similar trend observed for ASD (median difference 0.10 mm, p_FDR_ = 0.003).

No significant differences were observed between S-28 and R-28 across evaluated metrics, whereas S-multi showed significantly higher HD compared to S-full and R-28. Further details on site-specific segmentations, the evaluation of the in vivo trained model on synthetic ground truth, and additional mixtures of synthetic and in vivo-paired models are outlined in the Appendix.

### 3.2 Best-performing model evaluation

The following subsections analyze the S-full segmentation model.

#### 3.2.1 Radius analysis along the arch

**Fig. 4:**
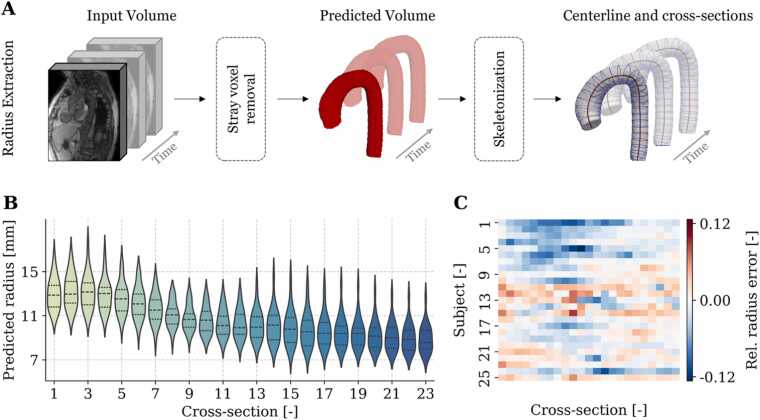
Radius analysis of the S-full predicted volumes. (A) Illustration of radius extraction, including outlier removal, surface generation, centerline, and cross-section extraction from 23 orthogonal and equidistant locations. (B) Violin plots of predicted peak-systolic radii along cross-sections along the arch. (C) Heatmap of relative radius errors of the S-full predicted test dataset for cross-section along the arch.

Fig. 4A illustrates the extraction of centerline and cross-sectional slices throughout the aortic arch from which time-resolved radii were computed. Panel B outlines violin plots of peak-systolic radii, averaged across the test dataset, and plotted for each cross-section along the centerline. Panel C shows a heatmap of peak-systolic relative radius errors, plotted for each subject of the test dataset and across the centerline’s cross-sections (horizontal axis). The resulting mean ± SD were found as (−0.9 ± 3.2)% with an absolute maximal value of 12.5%.

#### 3.2.2 Cross-section area analysis at PA-level

**Fig. 5:**
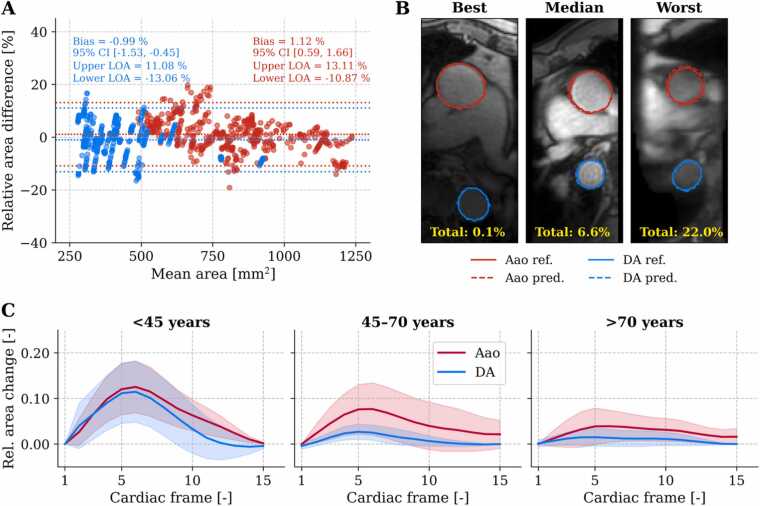
Evaluation of area prediction derived using the S-full model. (A) Bland-Altman plot of relative area errors in Aao (red) and DA (blue) region at the pulmonary artery cross-section across all cardiac frames, with bias and limits of agreement shown as dashed lines. (B) Representative cross-sections illustrating the best (left), median (middle), and worst (right) total area predictions, with predicted contours shown in dotted lines and reference contours in solid lines. (C) Relative area change over the cardiac cycle for the three age groups: younger than 45 years (left), 45 to 70 years (middle), and older than 70 years (right). *Aao* ascending aorta, *DA* descending aorta, *CI* confidence intervals, *LOA* limits of agreement.

Fig. 5 presents the cross-sectional area analysis derived from the binary mask of the predictions of the S-full model and the reference data, at the level of the PA. Panel A outlines Bland-Altman plots of relative area differences in ascending (Aao, red) and descending (DA, blue) aorta, yielding a small mean bias of 1.1% and −1.0% with LOA as [−10.9, 13.1]% and [−13.1, 11.1]% for Aao and DA, respectively. The CI were [0.6, 1.7]% and [−1.5, −0.5]%, respectively. Panel B outlines three examples of the test dataset with best (0.1%, left), median (6.6%, middle), and worst (22.0%, right) total relative area errors for combined Aao and DA regions. Fig. 5C shows relative area change across the cardiac cycle divided into three age groups (younger than 45 years, 45 to 70 years, and older than 70 years), with maximal variation found in the youngest group (Aao: (13.3 ± 4.9)%; DA: (13.2 ± 6.8)%) and progressively lower variation with age (mid-age to old: Aao (10.0 ± 3.6)%, DA (3.7 ± 1.6)% and oldest: Aao (4.9 ± 3.8)%, DA (2.5 ± 1.3)%).

Table 3 provides a quantitative summary of the temporally averaged segmentation metrics (DSC, IoU, Acc, Prec, Rec, HD, and ASD) of the S-full predicted against reference cross-sections, extracted at the PA-level.

**Table 3:** Temporally averaged distance metrics of the S-full model evaluated at single cross-sections in Aao and DA.

Segment	DSC ↑	IoU ↑	Acc ↑	Prec ↑	Rec ↑	HD (mm) ↓	ASD (mm) ↓
Aao	0.955±0.019	0.915±0.035	0.995±0.002	0.950±0.040	0.962±0.030	1.3±0.5	0.5±0.2
DA	0.961±0.021	0.926±0.038	0.997±0.002	0.962±0.031	0.962±0.036	1.1±0.4	0.3±0.2

*Aao* ascending aorta, *DA* descending aorta, *DSC* Dice similarity coefficient, *IoU* intersection over union, *Acc* accuracy, *Prec* precision, *Rec* recall, *HD* Hausdorff distance, *ASD* average surface distance

#### 3.2.3 Segmentation robustness

**Fig. 6:**
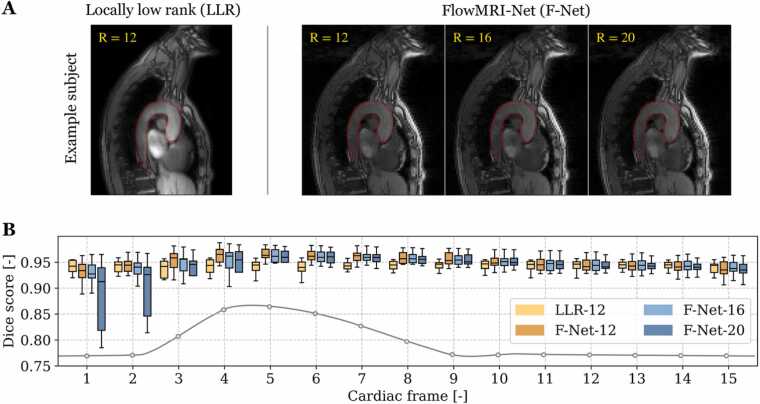
Evaluation of segmentation robustness depending on the reconstruction method and undersampling factor (R). (A) Representative data for R = 12 reconstructed with LLR (left) and with FlowMRI-Net (F-Net) for R = 12, 16, and 20 (right). (B) Temporal Dice scores for LLR (yellow) and F-Net (orange/blue) shown the first 15 cardiac frames with an example flow curve shown in gray. *LLR* locally low-rank regularization.

Robustness of the synthetically trained segmentation network was assessed in 10 subjects, reconstructed using two algorithms (LLR and FlowMRI-Net) and evaluated across multiple undersampling factors. Fig. 6 provides a qualitative overview. Panel A illustrates an example subject reconstructed with LLR at an acceleration factor of 12 (left) and FlowMRI-Net at acceleration factors of 12, 16, and 20 (from left to right), with predicted contours shown in red. Panel B displays time-resolved DSC of the first 15 cardiac frames, with boxplots presented alongside an example flow curve in gray. Results are shown for LLR-12 (yellow) and FlowMRI-Net (F-Net) at R = 12 (orange), R = 16 (light blue), and R = 20 (dark blue). Quantitative segmentation metrics are summarized in Table 4, demonstrating highest performance for data reconstructed with FlowMRI-Net at the lowest acceleration factor (F-Net (R = 12)) and lowest performance for the highest undersampling level (F-Net (R = 20)).

**Table 4:** Temporally averaged distance metrics for the S-full model, evaluated on data reconstruction using LLR and FlowMRI-Net (F-Net), for acceleration factors of 12, 16, and 20.

R-factor	DSC ↑	IoU ↑	Acc ↑	Prec ↑	Rec ↑	HD (mm) ↓	ASD (mm) ↓
LLR (R=12)	0.942±0.012	0.891±0.020	0.997±0.001	0.969±0.021	0.917±0.025	2.1±1.5	0.7±0.2
F-Net (R=12)	**0.952±0.018**	**0.908±0.032**	**0.998±0.001**	**0.937±0.026**	**0.967±0.020**	**1.9±1.7**	**0.6±0.2**
F-Net (R=16)	0.950±0.019	0.905±0.034	0.998±0.001	0.937±0.025	0.963±0.024	2.4±3.9	0.6±0.3
F-Net (R=20)	0.942±0.034	0.892±0.057	0.997±0.001	0.938±0.027	0.948±0.051	4.7±10.3	09±1.0

*LLR* locally low-rank regularization, *DSC* Dice similarity coefficient, *IoU* intersection over union, *Acc* accuracy, *Prec* precision, *Rec* recall, *HD* Hausdorff distance, *ASD* average surface distance

## 4 Discussion

We demonstrate that synthetically generated 4D flow CMR data can be effectively used to train the nnU-Net for time-resolved aortic segmentation, achieving high accuracy across in vivo datasets acquired with heterogeneous protocols. The best performance was obtained with the S-full model, which achieved a global DSC exceeding 95%, an IoU of 91%, and high accuracy (99.7%), precision (95.2%), and recall (96.0%), together with a mean HD of 1.7 mm and ASD of 0.6 mm, both well below image resolution.

Distance metrics were computed after anatomical boundary alignment through clipping, at inlet and outlet, to avoid penalizing predictions beyond the annotated region.

All methods, with the exception of S-multi, demonstrated robust performance, which we attribute in part to the inherent generalizability of the nnU-Net framework. We attribute the improved performance of our approach, despite a smaller training cohort relative to previous studies [14], [16], to the incorporation of temporal information and inclusion of diastolic frames with reduced vessel-to-background contrast, thereby increasing feature diversity. Importantly, S-28 and R-28 performed comparably and both outperformed S-multi, despite being trained on an identical number of cases. Given the reduced number of distinct anatomical backgrounds in S-multi, this suggests that anatomical diversity in backgrounds, even in modestly sized cohorts, substantially improves model generalization.

Although segmentation was performed using magnitude images only, the CFD simulations were essential for generating physically consistent synthetic 4D flow CMR data. In 4D flow CMR, the magnitude signal is influenced by the underlying velocity field and its interaction with VENC. Thus, realistic geometry-flow combinations are required for faithful image synthesis. In additional experiments, we replaced CFD-derived velocity fields with random flow patterns while keeping geometry and background unchanged. This resulted in a marked performance decline, indicating that coherent geometry-flow relationships are crucial for effective training.

Statistical analysis revealed significant overall differences between models. Although the performance gains of S-full over R-28 were modest within an already high accuracy range, small improvements in boundary precision remain relevant, as derived metrics such as wall shear stress are sensitive to subtle surface deviations [35]. No significant differences were observed between S-28 and R-28, indicating that purely synthetic training achieves performance comparable to in vivo data. Although S-multi demonstrated reduced stability, overall accuracy remained acceptable, indicating that even limited synthetic training data can provide a practical starting point for segmentation, particularly when annotated in vivo data are scarce.

The performance gap between S-28 and S-full further underscores the importance of anatomical variability. Although these datasets shared the same original number of distinct background anatomies, S-full included a broader range of aortic geometries, resulting in improved stability. This observation aligns with previous findings [19] demonstrating that body-to-aorta size influences segmentation performance. While a systematic analysis was not conducted, we generated a wide range of synthetic aortic geometries, effectively diversifying the training dataset. Taken together, these results highlight the critical importance of anatomically diverse training data, a factor that can be effectively controlled and expanded through data synthesis.

Quantitative evaluation of the S-full model revealed high consistency in radius and area estimates across the aortic arch and at the PA-level. Extracted radii showed the expected gradual reduction along the arch [36]. Relative radius errors exhibited a small bias (−0.9 ± 3.2)%, with slightly larger deviations in the ascending-to-top aortic region, as depicted in Fig. 4C. Although the maximum absolute relative radius error (∼13%) appears substantial, these deviations remain within image resolution.

Cross-sectional area analysis at the PA-level demonstrated strong agreement with reference segmentations. Bland-Altman analysis showed minimal bias (1% in the ascending and −1% descending aorta), with LOA spanning to approximately 13% in both regions.

Even the largest cross-sectional area deviation (∼22%) remained within the expected image resolution-related uncertainty.

The group-based analysis was included to demonstrate a potential downstream application of the segmentation framework. Geometric parameters derived from aortic segmentations, such as diameter and strain, are known to vary with age. Observing consistent trends within our cohort provided indirect evidence that the segmentation accuracy is sufficient to support meaningful quantitative analysis. Relative area variation decreased progressively with age (Aao: ∼13% in younger adults, ∼10% in mid-to-older aged subjects, and ∼5% in older adults), consistent with age-related vascular stiffening [37] and previously reported diameter changes [7]. These findings reflect the capability of the segmentation framework to preserve physiologically plausible trends; however, we emphasize that these observations reflect the accuracy characteristics of the segmentation model rather than physiological effects, and note that clinical conclusions cannot be drawn without evaluation in larger, age-, and sex-matched cohorts.

Finally, segmentation performance showed only limited sensitivity to the choice of reconstruction algorithm, but was affected by the degree of undersampling, consistent with the expected gradual loss of high-frequency spatial information and increased spatio-temporal smoothing at higher acceleration factors. For equivalent acceleration factors, FlowMRI-Net yielded slightly higher mean scores than LLR, although these differences were not statistically significant for DSC and IoU. At the highest acceleration factor, segmentation quality declined noticeably, reflected in substantially increased errors across evaluated metrics.

The pretrained S-full segmentation model, together with an illustrative example subject, is available at https://gitlab.ethz.ch/ibt-cmr/publications/4dflowmri-segmentation.

## 5 Limitations

Although data from multiple sites were included, the overall cohort size remains limited and covers a restricted range of pathologies. As a result, the generalization of the synthetically trained network cannot yet be fully established. However, the proposed framework can be readily extended by generating additional synthetic cases or incorporating in vivo data representing specific clinical conditions.

The synthetic training data were derived from a single field strength. Although model performance on lower field strengths was robust, differences in image characteristics across field strengths may affect generalization. Future studies incorporating multi-field-strength synthetic data may further improve robustness.

In this study, the aortic root and supra-aortic vessels were excluded, as they were not represented in the synthetic dataset. While inclusion is feasible through retraining with appropriate annotations, realistic simulations of the aortic root remain challenging.

Finally, synthetic data generation is computationally expensive. Although simpler deformation-based augmentation strategies were explored, they did not achieve comparable performance. Therefore, the development of more computationally efficient synthetic data generation methods remains critical, as presented in [38], [39], [40] and could be explored in future work.

## 5 Conclusion

Synthetic 4D flow CMR enables robust, time-resolved aortic segmentation of in vivo 4D flow CMR data, without manual annotation and with quantitative evaluation against known ground truth.

## Funding

This work was financially supported by grant 325230_197702 of the 10.13039/501100001711Swiss National Science Foundation (SNSF) and a Microsoft Joint Swiss Research grant.

## Author contributions

**Gloria Wolkerstorfer:** Writing – review & editing, Writing – original draft, Visualization, Validation, Software, Methodology, Investigation, Formal analysis, Data curation, Conceptualization. **Pietro Dirix:** Writing – review & editing, Data curation. **Cosima Jahnke:** Writing – review & editing, Resources. **Ingo Paetsch:** Writing – review & editing, Resources. **Robert Manka:** Writing – review & editing, Resources. **Stefano Buoso:** Writing – review & editing, Writing – original draft, Supervision, Methodology, Conceptualization. **Sebastian Kozerke:** Writing – review & editing, Writing – original draft, Supervision, Resources, Project administration, Methodology, Funding acquisition, Conceptualization.

## Declaration of competing interests

The authors declare that they have no known competing financial interests or personal relationships that could have appeared to influence the work reported in this paper.

## Figures and Tables

**Fig. A1:**
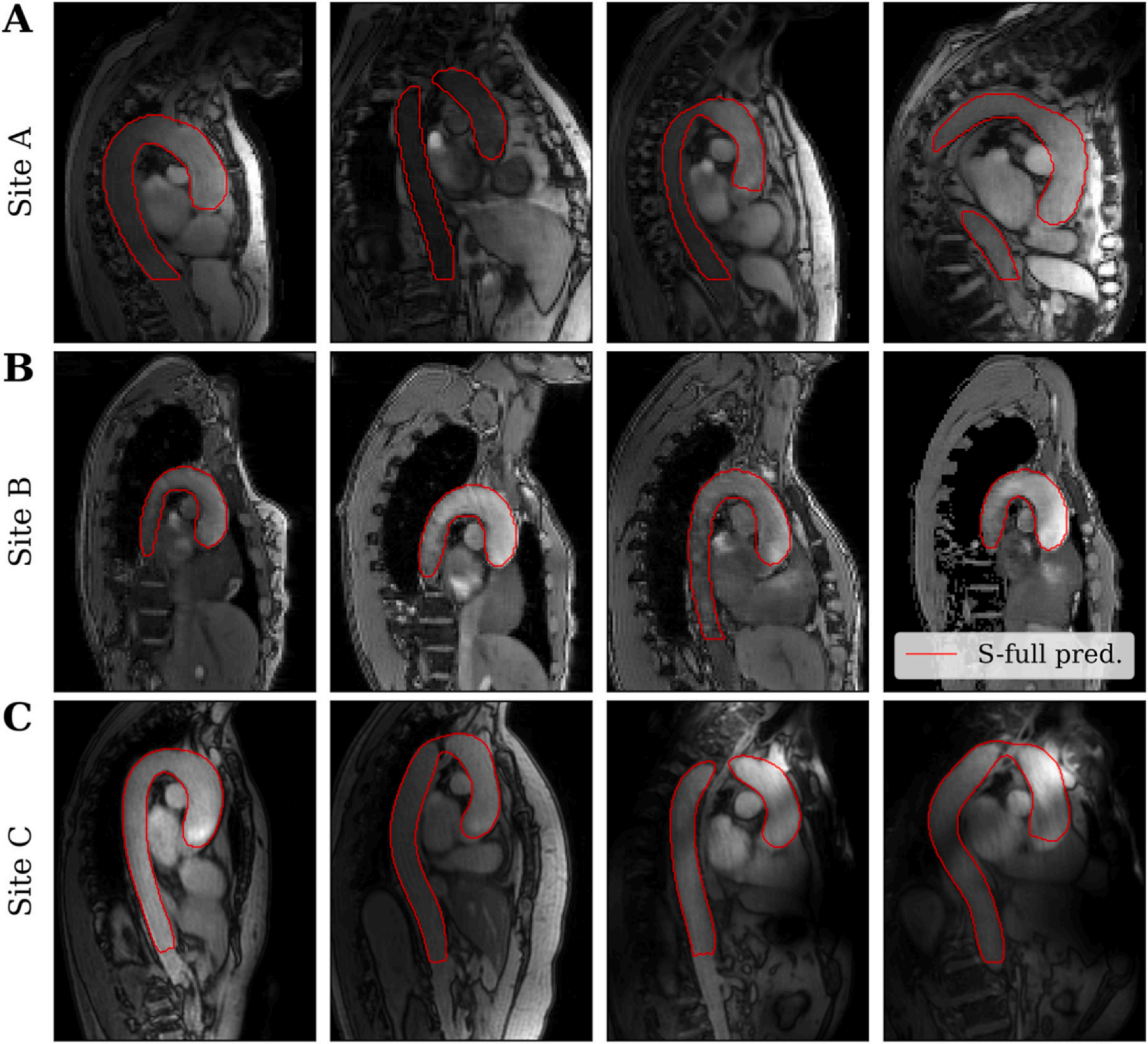
Peak-systolic cross sections of four representative subjects from each site (A-C), with S-full model predicted contours depicted in red.

**Fig. A2:**
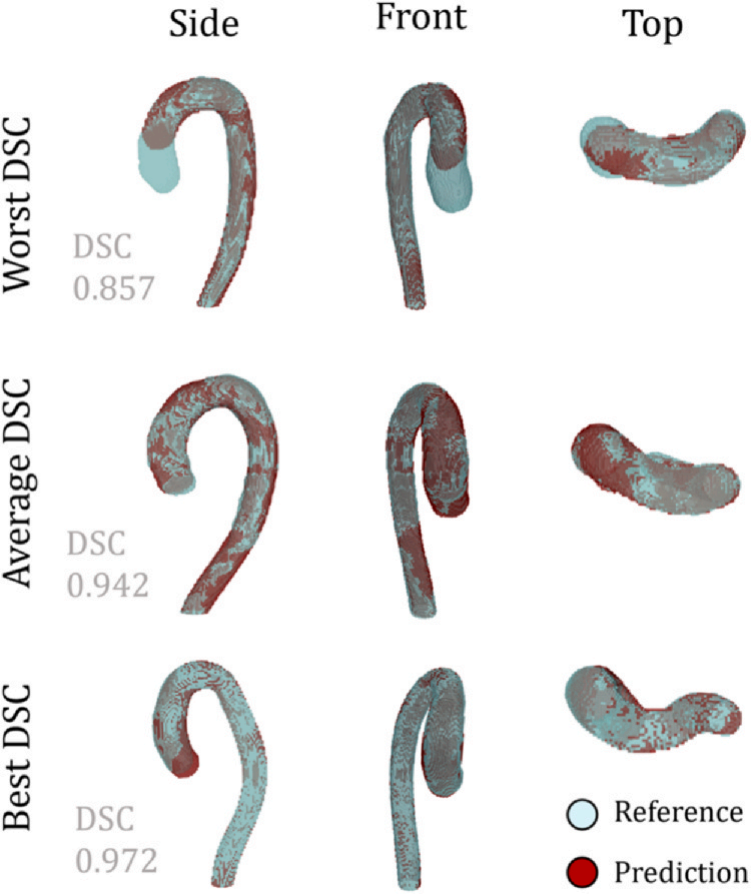
Examples of best, average, and worst frame volumes of the test dataset with reference masks in light blue and S-full predictions in red.

**Fig. A3:**
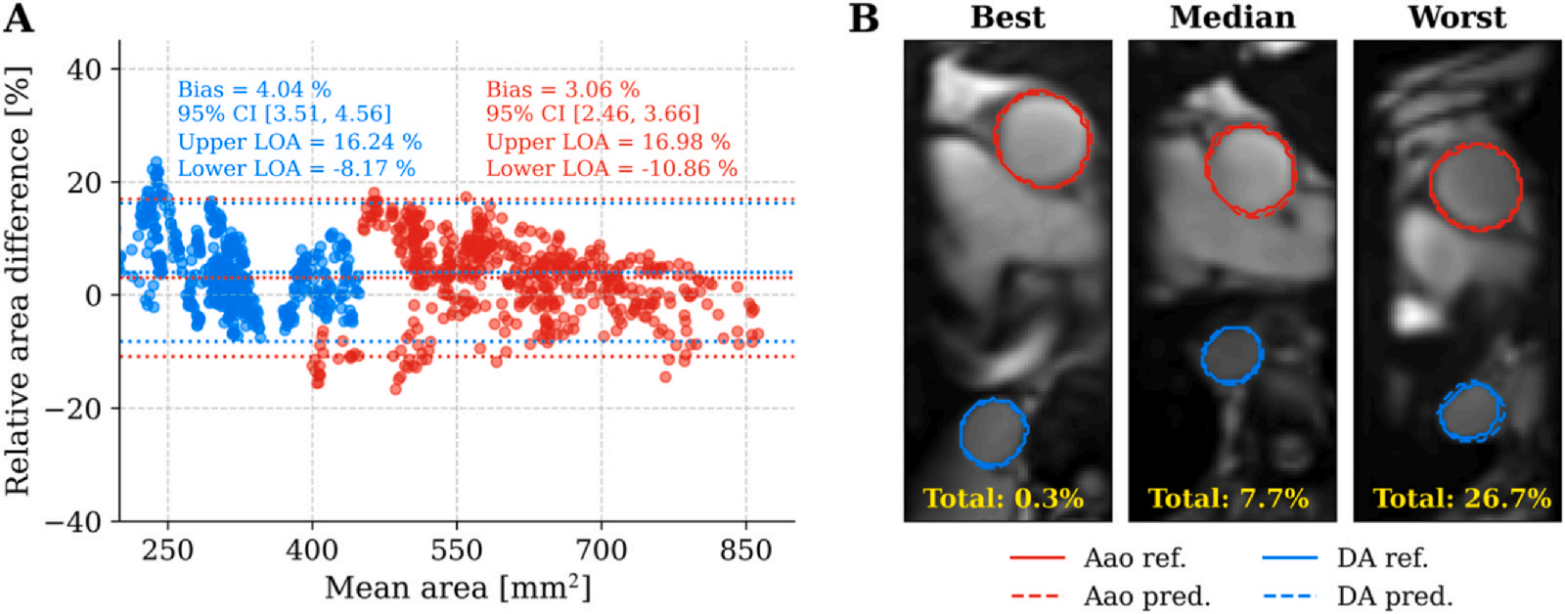
Area prediction with the R-28 model on synthetic data (S-28). (A) Bland-Altman plot of relative area errors in Aao (red) and DA (blue) region at the pulmonary artery cross-section across all cardiac frames, with bias and limits of agreement shown as dashed lines. (B) Representative cross-sections illustrating the best (left), median (middle), and worst (right) total area predictions, with predicted contours shown in dotted lines and reference contours in solid lines. *Aao* ascending aorta, *DA* descending aorta.

**Table A1:** Temporally averaged segmentation metrics stratified across acquisition sites A to C.

S-full pred	DSC ↑	IoU ↑	Acc ↑	Prec ↑	Rec ↑	HD (mm) ↓	ASD (mm) ↓
Site A	0.939±0.011	0.885±0.019	0.994±0.002	0.956±0.017	0.924±0.026	2.553±1.952	0.857±0.175
Site B	0.952±0.018	0.908±0.032	0.998±0.001	0.937±0.026	0.967±0.020	1.865±1.663	0.597±0.194
Site C	0.967±0.008	0.937±0.015	0.998±0.000	0.966±0.018	0.969±0.013	1.145±0.284	0.420±0.080

*DSC* Dice score, *IoU* intersection over union, *Acc* accuracy, *Prec* precision, *Rec* recall, *HD* Hausdorff distance, *ASD* average surface distance

**Table A2:** Temporally averaged distance metrics for the in-vivo R-28 model, evaluated on the synthetic S-28 data.

R-28 pred	DSC ↑	IoU ↑	Acc ↑	Prec ↑	Rec ↑	HD (mm) ↓	ASD (mm) ↓
Synthetic data	0.939±0.014	0.885±0.025	0.995±0.001	0.930±0.025	0.950±0.030	2.242±1.372	0.823±0.176

*DSC* Dice score, *IoU* intersection over union, *Acc* accuracy, *Prec* precision, *Rec* recall, *HD* Hausdorff distance, *ASD* average surface distance

**Table A3:** Temporally averaged distance metrics for additional combinations of R-28, S-28, S-multi, and S-full models.

Model	DSC ↑	IoU ↑	Acc ↑	Prec ↑	Rec ↑	HD (mm) ↓	ASD (mm) ↓
R-28+S-multi	0.953±0.018	0.910±0.032	**0.997±0.001**	0.952±0.026	0.954±0.025	1.892±2.504	0.613±0.235
R-28+S-28	0.954±0.018	0.912±0.032	0.997±0.002	0.952±0.024	0.956±0.026	1.796±2.349	0.592±0.226
R-28+S-full	0.953±0.022	0.911±0.038	0.997±0.002	0.954±0.023	0.953±0.033	1.800±2.352	0.584±0.237
S-full	**0.956±0.017**	**0.915±0.031**	0.997±0.002	**0.952±0.025**	**0.960±0.025**	**1.672±1.244**	**0.574±0.211**

*DSC* Dice score, *IoU* intersection over union, *Acc* accuracy, *Prec* precision, *Rec* recall, *HD* Hausdorff distance, *ASD* average surface distance

## Appendix A

### Synthetic model evaluation across sites

Table A1 summarizes segmentation metrics across individual sites for the S-full model. Segmentation results from sites B and C showed higher agreement across metrics, with best performance achieved with the contrast-enhanced dataset, which is expected due to the higher vessel-to-background contrast. This suggests that inference on contrast-enhanced data using models trained on non-contrast-enhanced data is feasible. The higher performance on site B relative to site A may reflect differences in signal-to-noise ratio at 1.5T and is further influenced by the limited size of the site A test set, which comprised only five subjects. Fig. A1 illustrates animations of four representative subjects from each site (A-C), with S-full segmentation contours depicted in red. Additionally, best, average, and worst predicted frames across all three datasets with reference data (light blue) and S-full model predictions (red) are displayed in Fig. A2.

### Segmentation accuracy and precision on synthetic ground truth

To analyze segmentation accuracy and precision relative to ground truth, we analyzed the in vivo trained model R-28 on synthetic 4D flow MRI data. Results of the averaged R-28 predictions on the S-28 dataset are outlined in Table A2.

Fig. A3 presents the cross-sectional area analysis derived from predictions with the R-28 model relative to ground-truth synthetic data, at the level of the PA. Panel A provides Bland-Altman plots of relative area differences in ascending (Aao, red) and descending (DA, blue) aorta, yielding a mean bias of 3.1% and 4.0% with LOA as [−10.9, 17.0]% and [−8.2, 16.2]% for Aao and DA, respectively. The CI were [2.5, 3.7]% and [3.5, 4.6]%, respectively. Panel B shows three examples of the synthetic dataset with best (0.3%, left), median (7.7%, middle), and worst (26.7%, right) total relative area error for combined Aao and DA regions.

### Additional combinations of in vivo and synthetic data models

All additional combinations of in vivo-paired with-synthetic models achieved similarly high frame-wise segmentation performance across all metrics, with overlapping central tendencies and small variances, see Table A3. DSC ranged from 0.953 to 0.945, IoU from 0.910 to 0.915, and frame accuracy was uniformly high (0.997 ± 0.002). While pairwise statistical tests identified significant differences for several comparisons, absolute performance differences were small, indicating only incremental improvements. Overall, the results suggest that the proposed model variants are largely equivalent in practical performance.

### Statistical Analysis

To quantify the performance of binary segmentation masks, the number of true positives (TP), false positives (FP), true negatives (TN), and false negatives (FN) was used to compute the following voxel-wise metrics:

• Dice Score (DSC) quantifies the spatial overlap between predicted and reference segmentations:
  $$\mathrm{Dice}=\frac{2\mathrm{TP}}{2\mathrm{TP}+\mathrm{FP}+\mathrm{FN}}$$
  ranging from 0 (no overlap) to 1 (perfect agreement).
• Intersection over Union (IoU) measures the ratio of the intersection to union:
  $$\mathrm{IoU}=\frac{\mathrm{TP}}{\mathrm{TP}+\mathrm{FP}+\mathrm{FN}}$$
  also ranging from 0 to 1, with stronger penalization for mismatches.
• Accuracy (Acc) quantifies the proportion of correctly classified voxels across the total dataset:
  $$\mathrm{Acc}=\frac{\mathrm{TP}+\mathrm{TN}}{\mathrm{TP}+\mathrm{TN}+\;\mathrm{FP}+\mathrm{FN}}.$$
• Precision (Prec) measures the reliability of positive predictions:
  $$\mathrm{Prec}=\frac{\mathrm{TP}}{\mathrm{TP}+\mathrm{FP}}$$
  with high values indicating few false-positive voxels.
• Recall (Rec), also known as sensitivity, quantifies the proportion of true positives detected:
  $$\mathrm{Rec}=\frac{\mathrm{TP}}{\mathrm{TP}+\mathrm{FN}}.$$
  For surface-based evaluation, let X and Y denote the sets of boundary points extracted from the predicted and reference segmentations, respectively, with d(x,y) defined as the Euclidean distance between points. Then the following point-set metrics can be defined:
• The Hausdorff distance (HD) quantifies the maximum surface discrepancy:
  HD(X,Y)=max(h(X,Y),h(Y,X))
  where h(A,B) is given by
  $$h(A,B)\;=\;\max_{a\in A}\left(\min_{b\in B}\left(d\left(a,b\right)\right)\right).$$
• The average surface distance (ASD) quantifies the mean bidirectional surface discrepancy as:

$$\mathrm{ASD}\left(X,Y\right)=\;\frac{1}{\left|X\right|+|Y|}\left(\sum_{x\in X}\underset{y\in Y}{\min(}d\left(x,y\right))+\sum_{y\in Y}\min_{x\in X}\left(d\left(x,y\right)\right)\;\;\right),$$

and is less sensitive to outliers than the HD.
